# Treatment for Hyperosmolar Dehydration in Hospitalised Adults: Protocol for a Scoping Review of Current Evidence and Gaps

**DOI:** 10.2196/87080

**Published:** 2026-06-19

**Authors:** Charlotte Henningsen Hansen, Linda Tram Mortensen, Birgitte Brandstrup, Anne Marie Beck, Stig Andersen, Mathias Brix Danielsen

**Affiliations:** 1 Department of Medicine, North Denmark Regional Hospital, Bispensgade 37, Hjørring, 9800, Denmark, 45 51866976; 2 Department of Surgery, Holbæk Sygehus, Holbæk, Denmark; 3 Department of Clinical Medicine, Faculty of Health and Medical Sciences, University of Copenhagen, Copenhagen, Denmark; 4 Unit for Dieticians and Nutrition Research, Copenhagen University Hospital – Herlev and Gentofte, Herlev Hospital, Herlev, Denmark; 5 Department of Geriatric Medicine, Aalborg University Hospital, Aalborg, Denmark; 6 Department of Clinical Medicine, Aalborg University, Aalborg, Denmark

**Keywords:** dehydration, fluid therapy, serum osmolarity, scoping review, geriatric medicine

## Abstract

**Background:**

Dehydration—specifically hyperosmolar dehydration (HD)—in adults is a common clinical condition affecting an estimated 25%‐58% of adults admitted to the hospital, depending on the method used to assess HD and the patient group. HD is defined as low total body water and increased serum osmolality over 300 mOsm/kg. Hypernatremia, increased blood urea nitrogen (BUN)/creatinine ratio, and calculated serum osmolarity are often used as proxy markers. The condition is associated with an increased risk of a range of complications, including increased morbidity and mortality. Despite the high prevalence, current treatment guidelines often fail to distinguish HD from hypovolemia. As a result, HD is often treated like hypovolemia with fluids containing isotonic saline rather than hypotonic fluids.

**Objective:**

The objective of this review is to identify and summarize existing studies on the treatment of HD in adults and to map the current evidence, highlight gaps in the literature and guide future research.

**Methods:**

The planned review will be conducted in accordance with the Preferred Reporting Items for Systematic Reviews and Meta-Analyses extension for Scoping Reviews (PRISMA-ScR) guidelines. A systematic search will be conducted in MEDLINE (PubMed), Embase (Ovid), Scopus, and the Cochrane Central Register of Controlled Trials, with additional examination of reference lists and citations (backward snowballing) and a search for completed trials based on found protocols (forward snowballing). Original studies of any design focusing on adult patients with a suspected low total body water and biochemically-confirmed HD receiving treatment for HD will be included. Studies will be excluded if: conducted on a non-adult population, no diagnoses of HD, focusing solely on hypovolaemia, or conducted in a non-hospital setting. Data charting will contain study characteristics, participant demographics, details of HD diagnosis, treatment approaches, and reported outcomes. Relevant information from the included studies will be reported in a narrative summary, supported by descriptive analyses of quantitative data where appropriate.

**Results:**

The primary search was completed in September 2025. An updated search will be performed before the completion of the review. As of April 2026, data extraction using Covidence on the relevant studies found in the primary search has been completed.

**Conclusions:**

This scoping review will provide an overview of the current evidence on the treatment of HD in patients referred to hospital, identifying key insights and evidence gaps to inform future research.

## Introduction

### Background

Hyperosmolar dehydration (HD) is defined by low total body water and elevated serum osmolality (above 300 mOsm/kg) and reflects a deficit in hypo-osmolar fluid most often as free water [1]. It differs from hypovolemia, which is a condition with a reduction in extracellular fluid volume (water and salt) [2,3]; HD is common in patients admitted to the hospital. Depending on the method of assessment, the reported prevalence of HD is 25%‐43% in admitted older adults [4,5], 58% in patients admitted with sepsis [6], and 36% in patients admitted with stroke [7]. Regardless of the patient group, HD is associated with adverse outcomes. This includes increased incidence of delirium, functional decline, prolonged hospital stays, higher readmission rates, and increased mortality [6,8,9].

Diagnosis of HD is challenging. Clinical signs such as reduced skin turgor and postural hypotension have poor sensitivity and specificity [10]. Direct measurement of serum osmolality is accurate but is often not routinely performed due to cost [11]. Both increased BUN (blood urea nitrogen) and the BUN/creatinine ratio are used as proxies for HD [11]. Increased sodium can also be used as a proxy measure, as it is a major contributor to serum osmolality; however, HD is independently associated with increased mortality even after adjusting for sodium [12,13], providing an indication that elevated sodium is only part of the problem. A validated osmolarity equation based on routine biochemistry (sodium, potassium, glucose, urea) offers a feasible screening alternative [14,15].

Current clinical guidelines often do not distinguish clearly between subtypes of low fluid status, such as HD and hypovolemia, resulting in uncertainty in treatment decisions [16,17]. Theoretically, HD represents a free water deficit and should be treated with oral water or hypotonic parenteral fluids like 5% glucose. Conversely, hypovolemia is generally managed with isotonic fluids [2]. Guidelines recommending treatment for HD lack solid references to support their recommendation [11,18,19]. The lack of clear differentiation and clinical evidence behind treatment recommendations in guidelines makes it challenging to implement targeted fluid therapy strategies for the treatment of HD [11,18-20].

Despite an increase in studies on HD [21], our preliminary search indicates a heterogeneous evidence base on the treatment of HD. Inconsistent definitions, diagnostic criteria, and outcome measures limit meaningful comparisons across studies and hinder the development of evidence-based treatment guidelines [11,18,22-24]. A scoping review is warranted to map the existing evidence and identify gaps in current knowledge.

### Objective

The objective is to identify and summarize existing studies on the treatment of HD in adults and to map the current evidence, highlight gaps in the literature, and guide future research.

## Method

This scoping review will be conducted in accordance with the JBI methodology for scoping reviews [25] and reported following the Preferred Reporting Items for Systematic Reviews and Meta-Analyses extension for Scoping Reviews (PRISMA-ScR) guidelines [26].

### Review Question

Which hydration treatments have been evaluated for adults with biochemically confirmed HD in a hospital setting?

### PCC Framework

The review question is structured using the PCC (Population, Concept, Context) framework as per the Joanna Briggs Institute (JBI) manual for scoping reviews. Table 1 presents the PCC elements and the corresponding sub-questions that will guide data extraction and synthesis.

**Table 1.** Review question based on the PCC (Population, Concept, Context) framework.

PCC element	Description	Sub-Questions
Population	Adults (minimum age 18 y) with hyperosmolar dehydration	What age groups are included in the studies? How is dehydration defined across studies? Are there differences in diagnosis between settings?
Concept	Hydration treatment (including oral, subcutaneous, or intravenous fluids)	What types of fluids are used? What is the route of administration? What are the reported outcomes (eg, symptom improvement, hospitalization, mortality)?
Context	Setting: hospital ward (any specialty), emergency department	In which setting is hydration therapy provided?

### Eligibility Criteria

We will include studies of adult populations admitted to the hospital with and treated for HD, regardless of admission diagnosis; HD must be biochemically confirmed.

Eligible concepts comprise treatment for HD, including oral, subcutaneous, or intravenous fluid therapy. Reports focusing solely on hypovolemia without laboratory evidence of hyperosmolarity will be excluded. Reports on patients without suspected reduced total body water will also be excluded (eg, iatrogenic hypernatremia).

Only studies conducted in hospital wards (any specialty) and emergency departments will be considered, irrespective of country or language.

We will include interventional, observational, qualitative studies, reviews, and case reports. Texts, opinion papers, and sources without direct patient data will be excluded.

### Exclusion Criteria

Studies will be excluded if they meet any of the following criteria:

1. Participants are children or adolescents (<18 y)
2. HD is not biochemically confirmed
3. Patients not suspected of having low total body water
4. Studies focusing on animals
5. Articles without primary patient data (eg, opinion papers, narrative reviews without original data)
6. Non-hospitalized populations
7. Full-text not available

### Information Sources

The following databases will be searched: MEDLINE (PubMed), Embase (Ovid), Scopus, and the Cochrane Central Register of Controlled Trials. Additional searches will include gray literature sources (eg, trial registries, dissertations, conference abstracts). Reference lists of included articles and relevant reviews will also be screened.

### Search Strategy

The search followed the three-step JBI approach. An initial exploration search in MEDLINE and Embase was conducted to identify keywords and index terms related to hyperosmolar dehydration and fluid therapy. These terms were combined into a comprehensive strategy and adapted for all databases. No restrictions on publication date or language were applied, and translation support was used when required. All search dates, strategies, and results have been documented. In addition, backward and forward snowballing was undertaken to identify further relevant studies beyond those retrieved in the initial database searches. The search strategy was developed in collaboration with a health sciences librarian, and the complete PubMed search string is presented in Table 2.

**Table 2.** Search string - PubMed.

No.	Search terms
#1	Search: “patients”[Mesh] OR “hospitalization”[Mesh] OR “aged”[Mesh] OR “aging”[Mesh] OR “geriatrics”[Mesh] OR patient*[Title/Abstract] OR inpatient*[Title/Abstract] OR outpatient*[Title/Abstract] OR hospital*[Title/Abstract] OR elder*[Title/Abstract] OR old adult*[Title/Abstract] OR older[Title/Abstract] OR geriatr*[Title/Abstract] OR aging[Title/Abstract] OR aging[Title/Abstract] Sort by: Publication Date
#2	Search: “Dehydration”[Mesh] OR dehydrat*[Title/Abstract] Sort by: Publication Date
#3	Search: “Fluid Therapy”[Mesh] OR fluid therap*[Title/Abstract] OR rehydrat*[Title/Abstract] OR iv fluid*[Title/Abstract] OR subcutaneous fluid*[Title/Abstract] OR hypodermoclys*[Title/Abstract] OR intravenous fluid*[Title/Abstract] OR parenteral fluid*[Title/Abstract] OR ((oral[Title/Abstract] OR orally[Title/Abstract]) AND fluid*[Title/Abstract]) OR hydration therap*[Title/Abstract] OR hydration treatment[Title/Abstract] Sort by: Publication Date
#4	Search: ((“Patients”[Mesh] OR “Hospitalization”[Mesh] OR “Aged”[Mesh] OR “Aging”[Mesh] OR “Geriatrics”[Mesh] OR patient*[Title/Abstract] OR inpatient*[Title/Abstract] OR outpatient*[Title/Abstract] OR hospital*[Title/Abstract] OR elder*[Title/Abstract] OR old 2434 adult*[Title/Abstract] OR older[Title/Abstract] OR geriatr*[Title/Abstract] OR aging[Title/Abstract] OR aging[Title/Abstract]) AND (“Dehydration”[Mesh] OR dehydrat*[Title/Abstract])) AND (“Fluid Therapy”[Mesh] OR fluid therap*[Title/Abstract] OR rehydrat*[Title/Abstract] OR iv fluid*[Title/Abstract] OR subcutaneous fluid*[Title/Abstract] OR hypodermoclys*[Title/Abstract] OR intravenous fluid*[Title/Abstract] OR parenteral fluid*[Title/Abstract] OR ((oral[Title/Abstract] OR orally[Title/Abstract]) AND fluid*[Title/Abstract]) OR hydration therap*[Title/Abstract] OR hydration treatment[Title/Abstract]) Sort by: Publication Date
#5	Search: ((“Adolescent”[Mesh] OR “Child”[Mesh] OR “Infant”[Mesh] OR “Pediatrics”[Mesh]) NOT “Adult”[Mesh]) OR ((pediatr*[Title] OR paediatr*[Title] OR child[Title] OR children[Title] OR newborn*[Title] OR neonat*[Title] OR infant*[Title] OR premature*[Title] OR preterm*[Title]) NOT adult*[Title]) Sort by: Publication Dat
#6	Search: “Animals”[Mesh] NOT “Humans”[Mesh] Sort by: Publication Date
#7	Search: ((((“Patients”[Mesh] OR “Hospitalization”[Mesh] OR “Aged”[Mesh] OR “Aging”[Mesh] OR “Geriatrics”[Mesh] OR patient*[Title/Abstract] OR inpatient*[Title/Abstract] OR outpatient*[Title/Abstract] OR hospital*[Title/Abstract] OR elder*[Title/Abstract] OR old adult*[Title/Abstract] OR older[Title/Abstract] OR geriatr*[Title/Abstract] OR aging[Title/Abstract] OR aging[Title/Abstract]) AND (“Dehydration”[Mesh] OR dehydrat*[Title/Abstract])) AND (“Fluid Therapy”[Mesh] OR fluid therap*[Title/Abstract] OR rehydrat*[Title/Abstract] OR iv fluid*[Title/Abstract] OR subcutaneous fluid*[Title/Abstract] OR hypodermoclys*[Title/Abstract] OR intravenous fluid*[Title/Abstract] OR parenteral fluid*[Title/Abstract] OR ((oral[Title/Abstract] OR orally[Title/Abstract]) AND fluid*[Title/Abstract]) OR hydration therap*[Title/Abstract] OR hydration treatment[Title/Abstract])) NOT (((“Adolescent”[Mesh] OR “Child”[Mesh] OR “Infant”[Mesh] OR “Pediatrics”[Mesh]) NOT “Adult”[Mesh]) OR ((pediatr*[Title] OR paediatr*[Title] OR child[Title] OR children[Title] OR newborn*[Title] OR neonat*[Title] OR infant*[Title] OR premature*[Title] OR preterm*[Title]) NOT adult*[Title]))) NOT (“Animals”[Mesh] NOT “Humans”[Mesh]) Sort by: Publication Date

### Selection of Studies

All retrieved references will be imported into Covidence (Covidence systematic review software, Veritas Health Innovation, Melbourne, Australia, accessed 2026), where duplicates will be removed. Prior to full screening, a pilot test on a small sample of studies will be conducted to ensure that both reviewers apply the eligibility criteria consistently. Following this, two reviewers will independently screen all titles and abstracts. Full texts of potentially eligible studies will then be assessed in duplicate, with reasons for exclusion documented. Disagreements will be resolved through discussion or, if necessary, by a third reviewer. The entire selection process will be illustrated in a PRISMA-ScR flow diagram.

### Data Extraction

Data will be extracted independently by two reviewers using a predefined charting form. Extracted variables will follow the PCC framework (Population, Concept, Context) and include study characteristics, participant demographics, diagnostic criteria, intervention details (type, route, dosage, setting), comparators (if any), outcomes (including biochemical, clinical, and health care utilization outcomes), and key findings. The predefined data extraction form is provided in Table 3.

**Table 3.** Data extraction form based on the PCC^a^ framework.

Author and year	Author and publication year
Country	Country where the study was conducted
Aim of Study	Stated aim or objective of the study
Study Design	eg, RCT^b^, observational, qualitative, review
Population	Description of study population
Age	Mean or median age, and age range if available
Dehydration Definition	Criteria used (including serum sodium, osmolality, osmolarity), including cutoff values when reported
Degree of hyperosmolarity	Mild, moderate or severe
Intervention Type and Details	Type of fluid used (including glucose, saline, Ringer’s lactate etc), administration route (including oral, intravenous, subcutaneous), volume, frequency, and administration context
Comparator (if any)	eg, standard care or alternative fluid treatment
Setting	eg, hospital ward (any specialty), emergency department
Outcomes	Categorized as biochemical, clinical and health care utilization outcomes (see data synthesis section for details)
Key Results	Summary of relevant findings related to hydration treatment

a PCC: Population, Concept, Context.

b Randomized controlled trial.

Variations in biochemical definitions and cutoff values of HD across studies will be extracted and reported as part of the evidence mapping.

As suggested by recent work [27], large language models such as ChatGPT-4o may also be explored as a supplementary tool to support the data extraction process. ChatGPT will only be used in addition to the two reviewers as a kind of third reviewer. Use and prompts will be at the discretion of the individual reviewer. All extracted data will be independently verified by human reviewers, and all final decisions will be made by the research team.

### Data Analysis and Data Synthesis

Findings will be summarized descriptively, focusing on the key outcomes identified in the PCC framework. Quantitative data, such as participant numbers, intervention types, and clinical outcomes, will be presented in tables to allow comparison across studies. Qualitative information, including study design and narrative outcomes, will be organized thematically.

The extracted data will be grouped into the following categories:

1. Population characteristics (age, setting)
2. Intervention characteristics (fluid type, route, amount, rate, comparators)
3. Outcomes, which will be further grouped into:
  - Biochemical outcomes (eg, serum sodium, osmolality, osmolarity, BUN/creatinine)
  - Clinical outcomes (markers of hydration status, mortality, symptom improvement)
  - Health care utilization outcomes (eg, length of hospital stay, readmissions)
  - Adverse effects of the treatment

These categories will be mapped to identify patterns and evidence gaps. Joanna Briggs Institute (JBI) scoping review methodology [25] and guidance from PRISMA-ScR [26] will be used to ensure a transparent synthesis. Narrative synthesis will be combined with visual presentation (charts, tables) where appropriate.

## Results

The initial literature search was completed on September 30, 2025, but will be repeated before publication of the review. Initial data extraction was finalized on April 16, 2026, using Covidence, resulting in the inclusion of 13 studies in this scoping review. We do not know whether repetition of the literature search will provide additional papers.

## Discussion

### Principal Findings

This scoping review aims to provide a comprehensive mapping of available evidence on hydration treatment in adults with HD in hospital settings. Based on preliminary searches, we expect the literature to be heterogeneous in terms of both the definition of HD and treatment approaches to the condition. Moreover, we anticipated that the evidence base was limited, especially regarding studies directly comparing different hydration strategies and their clinical outcomes. Thus, the review will highlight gaps in the literature that hinder standardization of clinical practice.

### Comparison to Prior Work

While dehydration in older adults has been widely studied and found to be associated with adverse clinical outcomes such as mortality, functional decline, and hospitalization [8,22-24,28], significantly fewer studies have focused specifically on HD as a separate clinical condition [18]. In clinical practice, and in current guidelines, HD is not consistently differentiated from other subtypes of fluid deficits, such as hypovolemia. This is especially evident in relation to treatment recommendations for HD [16,20]. This lack of clarity leads to variation in clinical practice and uncertainty about the choice of fluid. Mapping the existing evidence is therefore timely and relevant.

### Strengths and Limitations

This review has several important strengths. It will be conducted according to a predefined protocol and in accordance with the PRISMA-ScR guidelines, which support a systematic and transparent methodological framework. In addition, all relevant published literature on the topic will be considered, allowing for a comprehensive narrative synthesis of the available evidence. Nevertheless, some limitations must be acknowledged. Specifically, no formal risk-of-bias assessment will be performed for the included studies. Moreover, because the review is limited to biochemically verified HD in hospitalized adults, the findings may have limited applicability to patients in other clinical settings or children.

### Future Directions

By mapping available evidence and gaps in current knowledge, this review may help guide future interventional research, particularly in relation to standardized definitions, outcome measures, and treatment guidelines for HD.

### Dissemination Plan

The results of the review are expected to be disseminated through a peer-reviewed publication and in relevant clinical and academic forums. The aim is to ensure that the findings are accessible to both researchers and clinicians and may contribute to ongoing discussions about hydration management in hospitalized adults.

### Conclusion

This scoping review will map the available evidence on treatment approaches for HD in adult hospital and emergency patients. The findings will identify which therapeutic strategies have been studied, highlight inconsistencies in definitions and reporting, and reveal evidence gaps that can guide future research.

## Supplementary material

10.2196/87080 Checklist 1 PRISMA-ScR Checklist.

## Abbreviations

BUN: blood urea nitrogen
HD: hyperosmolar dehydration
JBI: Joanna Briggs Institute
PCC: Population, Concept, Context
PRISMA-ScR: Reporting Items for Systematic Reviews and Meta-Analyses extension for Scoping Reviews
RCT: randomized controlled trial
